# The Importance of Patient Systemic Health Status in High-Grade Chondrosarcoma Prognosis: A National Multicenter Study

**DOI:** 10.3390/cancers16203484

**Published:** 2024-10-14

**Authors:** Veroniek M. van Praag, Dominique Molenaar, Guus A. H. Tendijck, Gerard R. Schaap, Paul C. Jutte, Ingrid C. M. van der Geest, Marta Fiocco, Michiel A. J. van de Sande

**Affiliations:** 1 Department of Orthopedic Surgery, Leiden University Medical Center, Albinusdreef 2, 2333 ZA Leiden, The Netherlandsm.a.j.van_de_sande@lumc.nl (M.A.J.v.d.S.); 2 Department of Orthopedic Surgery, Amsterdam University Medical Center, Meibergdreef 9, 1105 AZ Amsterdam, The Netherlands; 3 Department of Orthopedic Surgery, University Medical Center Groningen, Hanzeplein 1, 9713 GZ Groningen, The Netherlands; 4 Department of Orthopedic Surgery, Radboud University Medical Center, Geert Grooteplein Zuid 10, 6525 GA Nijmegen, The Netherlands; ingrid.vandergeest@radboudumc.nl; 5 Mathematical Institute, Leiden University, 2333 CC Leiden, The Netherlands; 6 Department of Biomedical Science, Section Medical Statistics, Leiden University Medical Center, Albinusdreef 2, 2333 ZA Leiden, The Netherlands; 7 Princess Maxima Center for Pediatric Oncology, 3584 CX Utrecht, The Netherlands

**Keywords:** chondrosarcoma, ASA-score, patient systemic health, bone malignancies

## Abstract

**Simple Summary:**

This study investigated the impact of the general health status on the survival of patients with high-grade chondrosarcoma, a rare type of bone cancer. This systemic health status is often summarized using the American Society of Anesthesiologists (ASA) score. By analyzing data from 249 patients, the study found that a higher ASA score, older age, and more aggressive tumors were linked to poorer survival rates. This study highlights that a patient’s general health, especially for those with severe systemic diseases, should be considered when determining treatment plans, alongside traditional factors like tumor grade and age. These findings can improve decision-making for complex surgeries in patients with high-grade chondrosarcoma.

**Abstract:**

**Background:** Due to the relatively advanced age and high mortality rate of patients with high-grade chondrosarcoma (CS), it is important to holistically assess patient- and tumor characteristics in multidisciplinary team and shared decision-making with regard to treatment options. While current prognostic models include multiple tumor and treatment characteristics, the only patient characteristics that are commonly included are age and gender. Based on clinical experience, we believe that factors related to patient preoperative systemic health status such as the American Society of Anesthesiologists (ASA) score may be equally important prognostic factors for overall survival (OS). **Methods:** A retrospective nationwide cohort study was identified from four specialized bone sarcoma centers in The Netherlands. Patients with a primary CS grade II, III, and dedifferentiated CS were eligible. Prognostic factors including age at presentation, gender, ASA score, CVD, tobacco use, BMI, histological tumor grade, tumor size, pathological fracture, presentation after unplanned excision, type of surgery and surgical margin were evaluated. The outcome measure was OS at the time of surgery. The Kaplan–Meier methodology was employed to estimate OS; a log-rank test was used to assess the difference in survival. To study the impact of prognostic factors on OS, a multivariate Cox proportional hazard regression model was estimated. **Results:** In total, 249 patients were eligible for this study, and 89 were deceased at the end of follow-up. In multivariate analysis, histological grade (HR 2.247, 95% CI 1.334–3.783), ASA score III (HR 2.615, 95% CI 1.145–5.976, vs. ASA I), and age per year (HR: 1.025, 95% CI 1.004–1.045) were negatively associated with OS. No association was found between tobacco use, BMI, gender or cardiovascular disease and OS in this cohort. Pathological fracture and tumor size were only associated with OS in univariate analysis. **Conclusions:** This multicenter study is the first on sarcomas to include ASA in a prognostic model. Results show that ASA score as a proxy for patients’ systemic health status should be included when providing a prognosis for patients with a high-grade primary CS, besides the conventional risk factors such as tumor grade and age. Specifically, severe systemic disease (ASA score III) is a strong negative predictor. Conversely, we found no difference in OS between ASA scores I and II. These findings aid multidisciplinary team and shared decision-making with regard to these complex sarcoma patients that often require life-changing surgeries. **Level of Evidence:** Prognostic level III. See the instructions for authors for the complete description of levels of evidence.

## 1. Introduction

Chondrosarcoma (CS) is one of the most common bone malignancies in adults [1,2,3,4]. Primary or conventional CS, diagnosed as grade I to III, accounts for 20% of all bone malignancies and 90% of all CS. Dedifferentiated CS, also referred to as grade IV, accounts for around 10% of the CS [3,5,6]. Tumors with histological grade II, III and dedifferentiated are considered high-grade [7]. Reported 5-year survival rates are 74% (95% CI: 70–78%) for grade II, 31% (95% CI: 23–39%) for grade III and 28% (95% CI: 15–41%) for dedifferentiated [8,9,10,11,12,13].

Due to the relatively advanced age and high mortality rate of patients with high-grade CS, it is important to create a more holistic risk model including both tumor and patient characteristics e.g., systemic health status at diagnosis, as this could contribute to improving multidisciplinary team and shared decision-making. While current prognostic models include multiple tumor and treatment characteristics, the only patient characteristics that are commonly taken into account are age and gender [11,14,15]. Even in recent large prognostic models for other sarcoma types, no other patient factors are used [16,17]. 

The following patient systemic health status-related factors are well established as prognostic factors in other cancer types: American Society of Anesthesiologists (ASA) score, cardiovascular disease (CVD), Body Mass Index (BMI), and tobacco use [18,19,20,21,22,23,24,25]. ASA score has been shown to predict length of stay after cancer surgery [18,26] and impact the survival of patients with a range of different cancer types, such as upper tract urothelial cancer [27,28] and oral and oropharyngeal cancer [29]. Conversely, other studies did not find a significant association with survival in, e.g., gastric cancer patients [30] and head and neck cancer patients [31].

To our knowledge, these factors have never been evaluated for high-grade CS. Therefore, the objective of this study was to evaluate the (additional) prognostic value of patient systemic health status-related factors on OS of high-grade CS patients.

## 2. Methods

### 2.1. Study Design

Retrospective data were collected from all four specialized bone sarcoma centers in The Netherlands—the Leiden University Medical Center, the University Medical Center Groningen, the Amsterdam University Medical Center, and the University Medical Center Nijmegen. Almost all Dutch patients with CS are referred to these four hospitals; therefore, this study was set up as a nationwide multicenter retrospective cohort study [11]. All included centers follow the Oncoline treatment guidelines of the Netherlands Comprehensive Cancer Organization (IKNL).

### 2.2. Study Population

**Figure 1:**
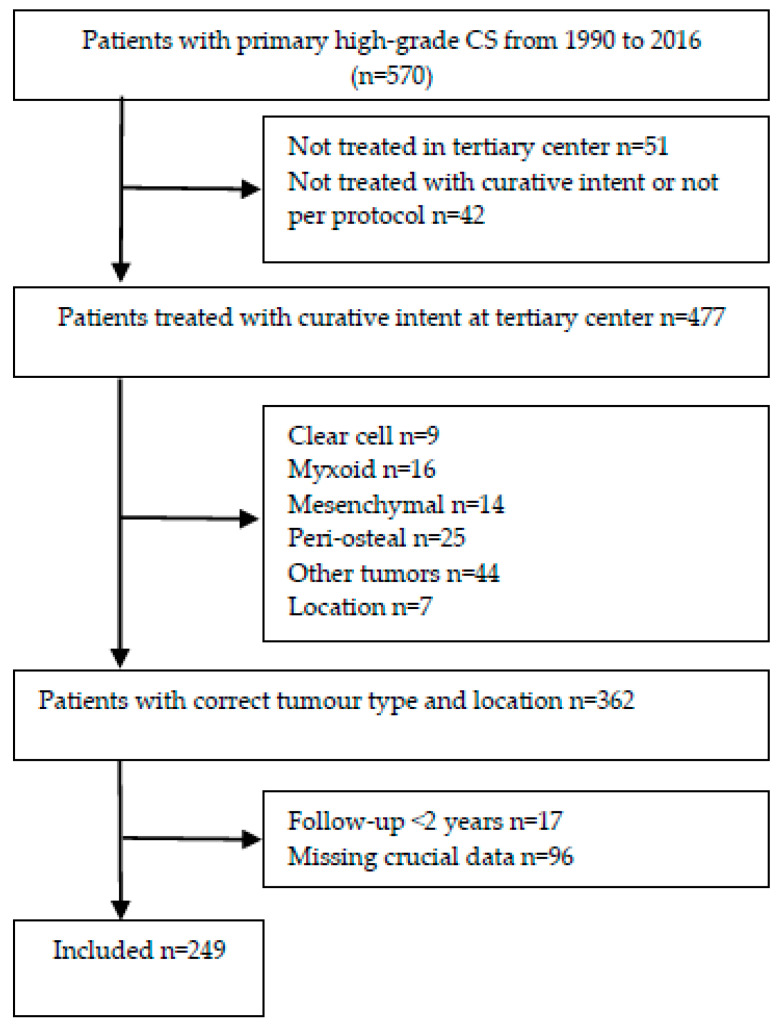
Flowchart showing patient inclusion and exclusion criteria and patient information.

Patients with a histologically proven conventional high-grade (grade II, III) or subtype dedifferentiated CS were recruited. Patients were primarily treated surgically, according to Dutch protocol, with curative intent between 1990 and 2016 at one of the four national centers for bone tumor treatment [32]. All patients had a follow-up duration of at least two years or experienced an event—local recurrence (LR), distant metastasis (DM) or death—within this period. Patients were considered ineligible if they presented with a DM or were diagnosed with DM within three months after diagnosis. This decision was made as the effect of DM at presentation (or so shortly after it that the DM was assumed to have already been present at presentation) on survival is known to be so strong that the patient’s systemic health status would be a much less relevant consideration. Tumors located above Th1 (including skull) or in the phalanges were also excluded. After excluding 632 patients with a low-grade tumor (grade I), a total of 570 patients were analyzed for eligibility and 249 met the inclusion criteria (Figure 1).

The mean age at presentation was 55 years (SD 17), with a slight male predominance (55.4%). Most patients were classified as either ASA I (45.6%) or II (46.2%). Many patients (45.7%) had a cardiovascular comorbidity and 28.3% were tobacco users. Median BMI was 26.0 (IQR 23.5–29.3) (Table 1).

**Figure A1:**
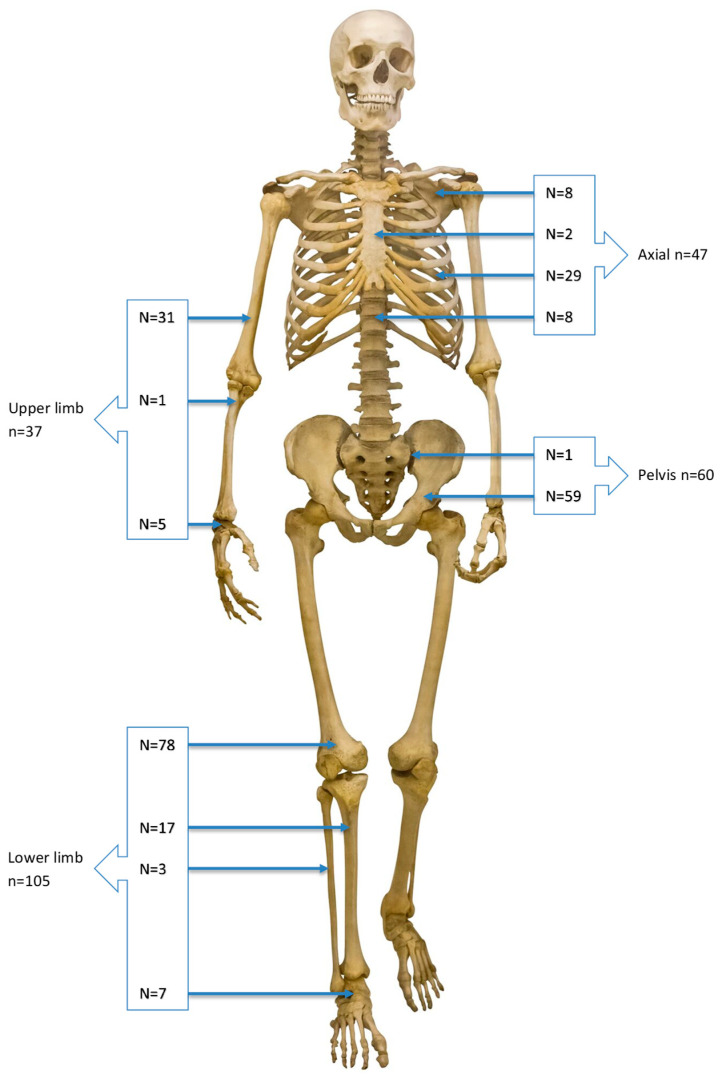
Overview of the Tumor Locations in the Study Population.

Forty-two percent of the tumors were in the lower extremity, 24% in the pelvis, 19% in the axial skeleton, and 15% in the upper extremity (Appendix A, Figure A1). Most patients (86.5%) experienced pain in the affected limb, and 11.5% had developed a pathological fracture due to the primary tumor. Grade II CS was the most common grade (76.7%), followed by dedifferentiated (12.4%) and grade III (10.8%) CS. Median tumor size was 8.2 cm (IQR 5.5–12.0). Seven percent of patients were referred after an unplanned excision elsewhere. Most patients (94%) were treated with wide excision of their CS (Table 1).

Over time, 75 patients (30.1%) developed an LR and 54 patients (21.7%) developed a DM. Four of the patients who developed a DM underwent metastasectomy of the lung with curative intent. Causes of death were due to sarcoma or other causes. At the final follow-up, 81% of all patients with a DM were deceased (Table 1).

### 2.3. Variables

To evaluate patients’ systemic health status, the ASA score was derived from the patient’s records as determined by the patient’s anesthesiologist at the time of surgery according to the definition of the ASA House of Delegates (Appendix B, Table A1) [33,34]. CVD, tobacco use and BMI were derived from patient records to the extent that relevant information was available. CVD was defined as any diagnosed disease related to the heart and vessels, including treated hypertension. It was decided that the cut-off value for non-smokers was set at smoking cessation for more than eight weeks prior to surgery. This choice was based on a meta-analysis by Wong et al., which concluded that respiratory complications after surgery for patients who stopped using tobacco for more than eight weeks before surgery were comparable to non-smokers [25]. No distinction was made between heavy and light smokers. 

Histological grade was based on pathology reports, which were all issued by sarcoma-specialized pathologists. For the prognostic model, it was decided to group histological grade III and subtype dedifferentiated (also mentioned in literature as type IV). Tumor size was defined as the largest dimension found in the pathological evaluation of the tumor. If this information was missing, size as described in the patient file or measured on the MRI was used. 

Surgical margins were derived from pathology reports. All included centers apply the WHO guidelines [3] for the classification of margins (intralesional: within lesion; marginal: within reactive zone-extracapsular; wide: beyond reactive zone through normal tissue within compartment).

### 2.4. Statistical Methods

Demographic and other baseline data including disease characteristics are listed and summarized with descriptive statistics. Categorical data are presented as contingency tables (frequencies and percentages). For continuous data, the mean and standard deviation are reported (Table 1).

To estimate the OS from the first surgery, Kaplan–Meijer’s methodology was employed. Patients alive at the end of follow-up were censored. The log-rank test was used to assess the difference between survival. Cox regression model was estimated to investigate the effect of risk factors on OS. In the multivariable Cox model, significant covariates from the univariate analysis were incorporated in the final model. Hazard ratios along with 95% confidence intervals are reported. Median follow-up was estimated with reverse Kaplan–Meier [35]. Statistical analyses were performed by using SPSS statistics 24; a *p*-value of less than 0.05 was considered significant.

An additional Cox regression model for local recurrence was estimated to investigate the effect of margins (free, marginal, intralesional), adjusted for tumor grade (II, III/dedif), tumor size (in cm) and adjuvant radiotherapy (yes/no). There was no violation of the proportional hazard assumption for each risk factor. The test of proportional hazards was related to time-weighted score tests of the proportional hazards hypothesis [36].

## 3. Results

**Figure 2:**
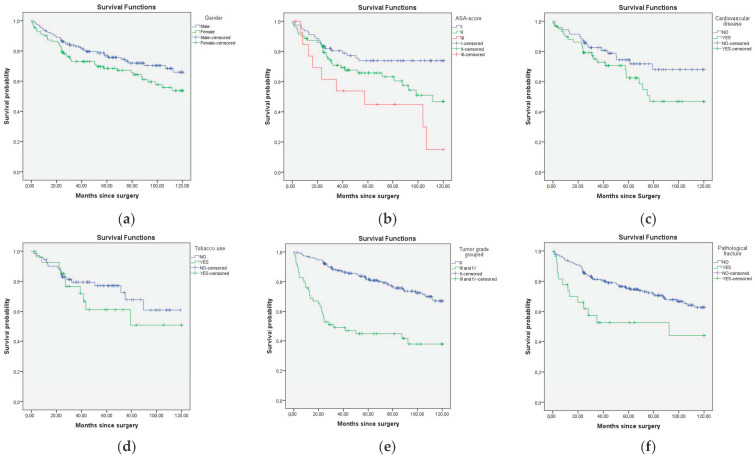
Kaplan–Meijer curves for overall survival for all prognostic factors: (**a**) Gender (*p* = 0.142), (**b**) ASA score (*p* = 0.001), (**c**) cardiovascular disease (*p* = 0.156), (**d**) tobacco use (*p* = 0.332), (**e**) tumor grade grouped (*p* < 0.001), (**f**) pathological fracture (*p* = 0.005).

The 5- and 10-year OS for grade II was 82% (95% CI 76–88%) and 67% (95% CI 58–76%), respectively, for grade III 52% (95% CI 32–72%) and 39% (95% CI 17–61%), respectively, and for grade differentiated, 39% (95% CI 29–56%) for both 5- and 10-year OS (Figure 2f). 

In this study, it was found that age at presentation (HR 1.036 per year; 95% CI 1.021–1.051), ASA score II (HR 1.840 95%; CI 1.072–3.158), ASA score III (HR 3.658 95%; CI 1.725–7.757), histological grade (HR 3.505 95%; CI 2.280–5.389), tumor size (HR 1.058 per cm 95%; CI 1.026–1.091) and pathological fracture (HR 2.217 95%; CI 1.259–3.904) were associated with OS in the univariate analysis, while gender, surgical margin, CVD, tobacco use, and BMI were not (Table 2).

Results from the multivariate Cox regression model show that age (HR: 1.025 per year; 95% CI 1.004–1.045), ASA score III (HR: 2.615; 95% CI 1.145–5.967) and histological grade (HR: 2.247; 95% CI 1.334–3.783) remained significantly associated with OS (Table 2). 

Results from the second multivariate Cox regression model show that surgical margins, i.e., marginal vs. intralesional (HR: 0.360; 95% CI 0.193–0.670) and free vs. intralesional (HR: 0.345; 95% CI 0.195–0.609), are significantly associated with LR.

## 4. Discussion

The main purpose of this study was to evaluate the (additional) prognostic value of patient systemic health status-related factors on the OS of high-grade CS patients. Our main finding is that ASA score, as a proxy for patient systemic health status, is a strong predictor for OS in high-grade CS patients. Specifically, severe systemic disease (ASA score III, HR 2.615 95% CI 1.145–5.976) is a strong negative predictor. 

In our analysis, we did not find a difference in OS between patients classified as ASA I (healthy) and ASA II (mild systemic disease). This finding suggests that there is no need to be more cautious about extensive surgery for patients classified as ASA II. 

Our dataset did not include patients with ASA status IV or V. Patients with these classifications are not considered fit for surgery in the centers that contributed to this study and would thus have been excluded based on the fact that they are not treated with curative intent. 

In our study, we also included the individual patient systemic health status factors BMI, tobacco use, and CVD. None of these factors had a significant effect on OS. Looking at the factors underlying the ASA classification, tobacco use, mild CVD and BMI < 40 would lead to an ASA II classification, which, as a classification, we also found did not significantly affect OS. In future studies, it would be interesting to collect the individual patient systemic health status factors that underly the ASA III classification (e.g., BMI > 40 or history of CAD/stents) to understand their individual effect on OS.

In this study, age and tumor grade were found to be strong independent predictors for OS, which is in line with the recent literature [8,14,37,38,39,40]. Tumor size was only found to be associated with OS in the univariate analysis. While some authors report no significant difference for size and OS [14,15], others do [11,38,39]. Fiorenza et al. [8] found tumor size to be an independent predictor. Pathological fracture was associated with worse survival in the univariate analysis. This result is in line with previously conducted studies, where pathological fractures were found to be of significant influence in the univariate and multivariate analysis [5]. Gender was not associated with OS in the univariate analysis, which is in line with most previous studies [14,37,38]. However, one SEER study by Nie et al. [39] found gender to be an independent prognostic factor for survival, with a higher risk for male patients in a multivariate Cox regression model. This result could possibly be caused by a much larger sample size compared to the other published papers, including this study.

In this study, the surgical margin did influence the risk of local recurrence but not overall survival. This is in line with existing literature [37,41,42], although a 2018 study by Stevenson et al. found that local recurrence did affect disease-specific survival [43]. 

In this study, only patients with a histological grade II, III or dedifferentiated CS were included. Most previous studies [13] also included grade I CS (now renamed ACT and considered locally aggressive and intermediate) and did not fully discriminate between the different grades [15,44,45]. However, patients with a grade I CS have an excellent reported prognosis [1,8]. Also, the percentages of the primary CS are 61%, 36%, and 3% for grades I, II, and III, respectively [3]. Consequently, including grade I for predicting OS in CS will, based on its survival rate and prevalence, disturb the outcome for high-grade lesions [15,44,45]. Therefore, this study only focused on high-grade CS to obtain more clinically relevant outcomes for the significant prognostic factors.

Patients with a non-primary tumor, those that developed a DM within three months of diagnosis and those treated without curative intent were excluded from this study to make the data set most suited to answering the research question. This subset of patients would be expected to have a higher survival rate than found in literature that includes the general patient population. This is indeed what we find: survival rates in this study are higher, especially those of patients with grade III or dedifferentiated tumors [8,9,10,11]. Therefore, the survival rates in this study should not be generalized beyond patients who meet the same inclusion criteria.

This study only looked at OS as an outcome metric. A patient’s systemic health status is also likely to impact longer-term health outcomes such as quality of life. The set-up of this study was not suited to investigating these outcomes, but this would be an interesting topic to further investigate in future studies.

Due to the retrospective nature of this study, some variables suffer from missing data. This is especially the case for BMI, CVD and smoking. The estimation of the Cox model has been performed on the complete case analysis. The relatively narrow 95% confidence intervals for the hazard ratios with the complete case analysis in the Cox model suggest the results are rather robust. However, the interpretation of the results that these individual factors are not significantly associated with survival should still be viewed in the context of the decreased power caused by the missing data.

To the best of our knowledge, we were the first to include ASA in our prognostic model. We suggest evaluating ASA as an additional prognostic factor for future models. In the shared decision-making, lifestyle changes where it seems accomplishable and the impact on the survival of the patient’s ASA status should be given proper attention and be made part of the multi-disciplinary approach. 

## 5. Conclusions

The ASA score as a proxy for patients’ systemic health status should be included when providing a prognosis for patients with a high-grade primary CS, besides the conventional risk factors such as tumor grade and age. This would aid the multidisciplinary team and shared decision-making in these complex sarcoma patients that often require life-changing surgeries.

## Figures and Tables

**Table 1:** Study characteristics.

Patient Characteristics	Values	% * (*n* = 249)
Age (years), mean (SD)	55 (17)	
Gender, male		55.4% (138)
ASA score (78 missing)		
I		45.6% (78)
II		46.2% (79)
III		8.2% (14)
Cardiovascular disease (120 missing)		45.7% (59)
Tobacco use (150 missing)		28.3% (28)
BMI, median (IQR) (150 missing)	26.0 (23.5–29.3)	
**Tumor characteristics**	**Values**	% * (*n* = 249)
Tumor grade (0 missing)		
II		76.7% (191)
III		10.8% (27)
Dedifferentiated		12.4% (31)
Tumor size (cm), median (IQR)	8.2 (5.5–12.0)	
Pathological fracture (15 missing)		11.5% (27)
Pain (56 missing)		86.5% (167)
**Treatment characteristics**	**Values**	% * (*n* = 249)
Presentation after unplanned excision (2 missing)		7.3% (18)
Type of surgery (3 missing)		
Curettage/excision with phenol		4.5% (11)
Curettage/excision without phenol		95.5% (235)
Surgical margin (11 missing)		
Free		46.2% (110)
Marginal		29.8% (71)
Intralesional		22.9% (57)
Local recurrence		30.1% (75)
Distant metastases		21.7% (54)
Outcome at last follow-up (0 missing)		
Alive with no evidence of disease		58.2% (145)
Alive with local recurrence		3.2% (8)
Alive with distant metastases		2.8% (7)
Died of disease		20.9% (52)
Died of other causes		12.4% (31)
Died of unknown cause		2.4% (6)

* Percentage without missing values; *n*, number; SD, standard deviation; ASA, American Society of Anesthesiologists; IQR, interquartile range; BMI, body mass index.

**Table 2:** Hazard ratio (HR) along with 95% confidence interval (CI) estimated with univariate and multivariate Cox regression model for overall survival.

	Univariate Analysis		Multivariate Analysis	
	HR (95% CI)	*p*-Value	HR (95% CI)	*p*-Value
Age (years)	1.036 (1.021–1.051)	**<0.001**	1.025 (1.004–1.045)	**0.017**
Gender, male	0.733 (0.483–1.112)	0.144		
ASA score				
I				
II	1.840 (1.072–3.158)	**0.027**	1.366 (0.741–2.517)	0.317
III	3.658 (1.725–7.757)	**<0.001**	2.615 (1.145–5.976)	**0.023**
Cardiovascular disease	1.549 (0.843–2.846)	0.156		
Tobacco use	1.463 (0.675–3.170)	0.335		
BMI	0.962 (0.891–1.040)	0.333		
Grade III/dediff vs. II	3.505 (2.280–5.389)	**<0.001**	2.247 (1.334–3.783)	**0.002**
Tumor size (cm)	1.058 (1.026–1.091)	**<0.001**	1.042 (1.000–1.086)	0.051
Pathological fracture	2.217 (1.259–3.904)	**0.006**		
Presentation after unplanned excision	0.738 (0.299–1.822)	0.511		
Type of surgery curettage vs. excision	2.796 (0.687–11.388)	0.151		
Surgical margin		0.940		
Free				
Marginal	1.050 (0.637–1.731)	0.849		
Intralesional	1.097 (0.649–1.855)	0.730		

All differences marked in bold are statistically significant at the 95% level.

## Data Availability

The data presented in this study are available upon request from the corresponding author due to patient privacy and legal restrictions.

## Appendix B

**Table A1:** ASA Physical Status Classification System.

	Definition	Examples (Including, but Not Limited To)
ASA I	A normal healthy patient	Healthy, non-smoking, no or minimal alcohol use
ASA II	A patient with mild systemic disease	Mild diseases only without substantive functional limitations. Examples include (but not limited to): current smoker, social alcohol drinker, pregnancy, obesity (30 < BMI < 40), well-controlled diabetic disease/hypertension, mild lung disease
ASA III	A patient with severe systemic disease	Substantive functional limitations; one or more moderate to severe diseases. Examples include (but not limited to): poorly controlled diabetic disease or hypertension, chronic obstructive pulmonary disease, morbid obesity (BMI ≥ 40), active hepatitis, alcohol dependence or abuse, implanted pacemaker, moderate reduction in ejection fraction, end-stage renal dysfunction (ESRD) undergoing regularly scheduled dialysis, premature infant post-conceptual age < 60 weeks, history (>3 months) of myocardial infarct (MI), cerebrovascular event (CVA), transient ischemic event (TIA), or coronary artery disease (CAD)/stents.
ASA IV	A patient with severe systemic disease that is a constant threat to life	Examples include (but not limited to): recent (<3 months) MI, CVA, TIA, or CAD/stents, ongoing cardiac ischemia or severe valve dysfunction, severe reduction in ejection fraction, sepsis, DIC, ARD or ESRD not undergoing regularly scheduled dialysis
ASA V	A moribund patient who is not expected to survive without the operation	Examples include (but not limited to): ruptured abdominal/thoracic aneurysm, massive trauma, intracranial bleed with mass effect, ischemic bowel in the face of significant cardiac pathology or multiple organ/system dysfunction
ASA VI	A declared brain-dead patient whose organs are being removed for donor purposes	

American Society of Anesthesiologists—ASA Physical Status Classification system [internet]. www.Asahq.org. (accessed on 13 December 2020). Available from: https://www.asahq.org/standards-and-practice-parameters/statement-on-asa-physical-status-classification-system, accessed on 13 July 2024.
